# The College of Nursing (Limited by Guarantee)

**Published:** 1920-10-09

**Authors:** 


### The College of Nursing
### (Limited by Guarantee).

### IRISH BRANCH.

A MEETING of College members will be held on Thursday, October 14, at 54 Fitzwilliam Square, Dublin, at 7.30 P.M., for the purpose of considering the desirability of forming a Local Centre for Dublin and district. Will any College member at present working in Dublin who has not received a notice of the meeting kindly communicate with the Secretary of the Irish Board at above address.

### EDINBURGH CENTRE.

Syllabus for 1920-1921:—November 17, "Public Speaking, and the Management of Meetings," Miss Alice Low, O.B.E. December 15, "Some Points in Fever Nursing," Claude B. Ker, M.D., F.R.C.P.E. January 19, "Dietetics in Disease," G. D. Mathewson, M.D., F.R.C.P.E. February 16, "On the Nature of Treatment of Functional Nervous Disorders, particularly in relations to those arising during the late War," Arthur Taylor, M.D., F.R.C.P.E. March 15, "The Nursing of Abdominal Cases," Alexander Miles, M.D., F.R.C.S.E. April 13, "The Modern Treatment of Venereal Diseases," Dr. Mary Macnicol. The lectures will be given on Wednesday afternoons, at 3.30, in the Nurses' Club, 8 Drumsheugh Gardens.

### BIRMINGHAM THREE COUNTIES CENTRE.

A MEETING will be held in the Lecture Theatre of the General Hospital, Birmingham (by kind permission of the Governors), on Saturday, October 9, at 3 P.M. Miss Sheriff-MacGregor, Organising Secretary to the College of Nursing, Ltd., will address the meeting.

### GENERAL NURSING COUNCIL.

A MEETING of the General Nursing Council will be held at 2 P.M. on Friday, October 15, 1920, in Room III., third floor, Ministry of Health, Whitehall.

